# ERAP1 genetic variations associated with HLA-B27 interaction and disease severity of syndesmophytes formation in Taiwanese ankylosing spondylitis

**DOI:** 10.1186/ar3855

**Published:** 2012-05-25

**Authors:** Chin-Man Wang, Huei-Huang Ho, Su-Wei Chang, Yeong-Jian Jan Wu, Jing-Chi Lin, Pi-Yueh Chang, Jianming Wu, Ji-Yih Chen

**Affiliations:** 1 Department of Physical Medicine and Rehabilitation, Chang Gung Memorial Hospital, Chang Gung University, College of Medicine, No. 5, Fu-Shin St. Kwei-Shan, Tao-Yuan, 33375 Taiwan; 2 Department of Medicine, Division of Allergy, Immunology and Rheumatology, Chang Gung Memorial Hospital, Chang Gung University, College of Medicine, No. 5, Fu-Shin St. Kwei-Shan, Tao-Yuan, 33375 Taiwan; 3 Clinical Informatics and Medical Statistics Research Center, Chang Gung University, 259 Wenhua 1st Road, Kwei-Shan, Tao-Yuan, 33375 Taiwan; 4 Department of Laboratory Medicine, Chang-Gung Memorial Hospital and Department of Medical Biotechnology and Laboratory Science, Chang Gung University, No. 5, Fu-Shin St. Kwei-Shan, Tao-Yuan, 33375 Taiwan; 5 Deptartment of Veterinary and Biomedical Sciences, 235B Animal Science/Vet. Med. Bldg., University of Minnesota,1988 Fitch Avenue, St. Paul, MN 55108, USA

## Abstract

**Introduction:**

Ankylosing spondylitis (AS) is a familial, heritable disease specified by syndesmophyte formation leading to an ankylosed spine. Endoplasmic reticulum aminopeptidase 1 (ERAP1) genetic variations have been widely proved to be associated with AS in several ethnic populations. The aim of this study was to investigate whether ERAP1 single nucleotide polymorphisms (SNPs) are associated with AS susceptibility and disease severity in Taiwanese.

**Methods:**

Four ERAP1 SNPs (rs27037, rs27980, rs27044 and rs30187) were genotyped in 797 Taiwanese AS patients and 1,150 healthy controls. Distributions of genotype and alleles were compared between AS patients and healthy controls, and among AS patients stratified by clinical parameters.

**Results:**

The SNP rs27037T allele appeared to be a risk factor for AS susceptibility (*P *= 5.5 × 10^-5^, OR 1.30, 95% CI: 1.15 to 1.48; GT+TT vs. GG *P *= 9.3 × 10^-5^, OR 1.49, 95% CI: 1.22 to 1.82). In addition, the coding SNP (cSNP) rs27044G allele (*P *= 1.5 × 10^-4^, OR 1.28, 95% CI: 1.13 to 1.46; CG+GG vs. CC, *P *= 1.7 × 10^-3^, OR 1.44, 95% CI: 1.15 to 1.81) and the cSNP rs30187T allele (*P *= 1.7 × 10^-3^, OR 1.23, 95% CI: 1.08 to 1.40; CT+TT vs. CC *P *= 6.1 × 10^-3^, OR 1.38, 95% CI: 1.10 to 1.74) were predisposing factors for AS. Notably, the rs27044G allele carriers (CG+GG vs. CC, *P *= 0.015, OR 1.59, 95% CI: 1.33 to 2.30) and rs30187T allele carriers (CT+TT vs. CC, *P *= 0.011, OR 1.63, 95% CI: 1.12 to 2.38) were susceptible to syndesmophyte formation in AS patients. Furthermore, two cSNPs (rs27044 and rs30187) strongly associated with HLA-B27 positivity in AS patients. Finally, the ERAP1 SNP haplotype TCG (rs27037T/rs27980C/rs27044G) is a major risk factor for AS (adjusted *P *<0.00001, OR 1.38, 95% CI: 1.12 to 1.58) in Taiwanese.

**Conclusions:**

This study provides the first evidence of ERAP1 SNPs involving syndesmophyte formation. The interactions between ERAP1 SNPs and HLA-B27 play critical roles in pMHC I pathway processing contributing to the pathogenesis of AS in multiple populations.

## Introduction

Ankylosing spondylitis (AS) is a chronic inflammatory arthritis that preferably affects the sacroiliac and spine joints in young males. Chronic inflammation in joints causes the alteration of joint architecture with new bone formations, and joint fusions may occur consequently [1,2]. The unique structural changes of syndesmophyte formation and ankylosis of the vertebrae are the primary causes of early severe work disability of AS patients during disease progressions [1-4]. Multiple factors and complex biological interactions may be involved in AS development [5-8].

Twin and family studies revealed a substantial proportion of heritability in AS susceptibility. Genetic studies indicate that HLA-B27 in the MHC (major histocompatibility complex) locus confers the greatest risk to AS susceptibility. However, genome wide association studies (GWAS) also reveal that the non-MHC genes contribute to the AS disease process [4,5,8-10]. Several genes and genetic regions have now been discovered and known to be associated with AS susceptibility and severity [8-10]. Of note, there were conflicting association results between Caucasians and Asians [9-12].

Endoplasmic reticulum aminopeptidase 1 (ERAP1) (also known as aminopeptidase regulator of TNFR1 shedding 1 or ARTS1) is an important non-MHC gene associated with AS in genetic studies [9,10]. Functionally, ERAP1 facilitates the antigen peptide loading onto the major histocompatibility complex class I (MHC I) through trimming the fragmented antigen peptides into the optimal length for peptide/MHC I (pMHC I) complex formation, which is necessary for effective immune responses [13-17]. In addition, ERAP1 involves the shedding of pro-inflammatory cytokine receptors for TNFα, IL-1, and IL-6 [18-20]. ERAP1 SNPs are associated with AS in several ethnic populations [9-12,21-26], but it is unknown whether ERAP1 SNPs have a role in AS development in Taiwanese. The aim of the present study was to examine whether the ERAP1 SNPs are associated with AS susceptibility and have effects on AS disease severity of syndesmophyte development in Taiwanese.

## Materials and methods

### Study subjects

The present study recruited 797 patients who fulfilled the 1984 revised New York diagnostic criteria for AS [27], and were followed up at Chang Gung Memorial Hospital (a 3,600-bed medical center and university hospital). Radiographs of the cervical, thoracic and lumbar spine were used by rheumatology specialists to evaluate syndesmophyte formations according to modified Stoke's Ankylosing Spondylitis Spinal Score (mSASSS). To ensure the accuracy of evaluation, two rheumatology specialists (Chen and Ho) independently scored the syndensmophyte formations by blindly reading radiographs of AS patients for appropriate inter- and intra-reader reliability. The X-ray observations were further classified into three groups: group 1 patients did not have any syndesmophyte formations (mSASSS <3), group 2 patients had less than four fused syndesmophyte formations (mSASSS <24) and group 3 patients had four or more syndesmophyte formations (mSASSS >24). The rare disagreements of the radiograph-based evaluations were resolved through consultations between two physicians to eliminate any subjectivity. HLA-B27 antigen positivity was determined by flow cytometry analysis and/or PCR assays. In this study, a total of 1,150 healthy normal controls (512 males and 638 females) were recruited, following a questionnaire survey to exclude donors with rheumatoid arthritis (RA), systemic lupus erythematous (SLE), AS and autoimmune thyroiditis, diabetes mellitus (DM), viral hepatitis (HBV and HCV) infections and cardiovascular diseases. The age of healthy control donors ranged from 18 to 64 years-old with a mean age of 40.3 ± 10.7. The local ethics committee of Chang Gung Memorial Hospital approved the present study. Informed consent was obtained from all patients

### Nucleic acid isolation

Anti-coagulated peripheral blood was obtained from healthy control donors and AS patients. Genomic DNA was isolated from EDTA anti-coagulated peripheral blood using the Puregene DNA isolation kit (Gentra Systems, Minneapolis, MN, USA) as previously described [28].

### TaqMan-based assays for ERAP1 SNPs

ERAP1 SNPs were genotyped with the TaqMan SNP Made to Order Assays from Applied Biosystems (ABI, Foster City, CA, USA). ERAP1 genotypes were determined using ABI TaqMan Genotyper software according to the vendor's instruction (Foster City, CA, USA).

### Statistical analysis

We carried out single-locus ERAP1 SNP analyses in 797 AS patients and 1,150 normal, healthy controls. Three chi-square tests (the genotype test, the allele test and Cochran-Armitage trend test) were performed. Associations of SNPs with AS (*P *<0.05) were identified using Plink [29] and the SAS/Genetics software package release 8.2 (SAS Institute, Cary, NC, USA). For the analysis of risk genotypes/alleles, logistic regression models adjusted for sex were used to calculate *P*-values, odds ratios (ORs) and their 95% confidence intervals. Linkage disequilibrium (LD) between marker loci was assessed and haplotype blocks were constructed using Haploview 4.1(Broad Institute of MIT, Cambridge, MA, USA). For each haplotype combination estimated, the haplotype-trait association was tested within different subgroups of disease status (case vs. control), HLA B27 positivity, and syndesmophyte formation were tested for the haplotype-trait association using the SAS HAPLOTYPE procedure. To investigate the association of SNPs with clinical characteristics, we controlled for each of clinical characteristics and performed logistic regression analyses. The 5% level of significance (*P *<0.05) was adopted for all the analyses. To account for the confounding effects between HLA-B27 positivity and syndesmophyte, we performed the stratified analysis with Cochran -Mantel -Haenszel (CMH) tests for ERAP1 SNP and syndesmophyte formation associations, controlling for HLA-B27 positivity. The results of the trend test, genotype and allelic analyses were adjusted for HLA-B27 positivity. We also carried out analyses with CMH tests for ERAP1 SNP and HLA-B27 associations, adjusted by syndesmophytes formation. The *P*-values, ORs and 95% CIs for the trend test, genotype and allelic analyses adjusted for syndesmophyte formation.

## Results

### Clinical characteristics of AS study cohort

The present study recruited 797 Taiwanese AS patients (667 males and 130 females). The onset ages of 697 AS patients ranged from 5 to 60 years-old. The onset age of a single female was 72 years-old. We were unable to identify exact onset ages of the other 99 AS patients in the study. Among AS patients, 739 (92.7%) patients were HLA-B27 carriers and 393 patients demonstrated syndesmophyte formation (group 2 with mSASSS <24 plus group 3 with mSASSS >24) based on spine radiograph data. Syndesmophyte formation was significantly more common in AS patients positive for HLA-B27 (380 out of 739, 51.4%) as compared to AS patients negative for HLA-B27 (13 of 58, 22.4%) (*P *<0.0001, OR 3.66, 95% CI: 1.94 to 6.91). Syndesmophyte formation was also significantly enriched in male AS patients (55%, 367 of 667) as compared to the female AS patients (20%, 26 of 130) (*P *<0.0001, OR 4.89, 95% CI: 3.10 to 7.72). Thus, HLA-B27 positivity and male gender were two major risk factors for AS syndesmophyte formation in our cohort. As shown in Table 1, most AS patients in this cohort had long disease courses (≥5 years) whereas patients with longer disease durations (>20 years) were enriched in AS with the syndesmophyte formation group (54.5%), indicating that the disease duration is also a risk factor for syndesmophyte formation.

**Table 1:** The demographic and clinical characteristics of AS patients positive and negative for sydesmophyte

Clinical characteristic	AS N = 797 (%)	Sydesmophyte positive N = 393 (%)	Sydesmophyte negative N = 404 (%)
**Gender (female)**			
	130 (16.3%)	26 (15.1%)	104 (25.7%)
**Age at onset**			
≤16	117 (14.7%)	55 (14.0%)	62 (15.4%)
17 to 40	533 (66.9%)	274 (69.7%)	259 (64.1%)
41 to 60	47 (5.9%)	12 (3.1%)	35 (8.7%)
>60	1 (0.1%)	0 (0%)	1 (0.2%)
Undetermined	99 (12.4%)	52 (13.2%)	47 (11.6%)
**Disease duration**			
<5 years	20 (2.5%)	4 (1.0%)	16 (4.0%)
5 to 10 years	118 (14.8%)	17 (4.3%)	101 (25.0%)
11 to 15 years	134 (16.8%)	54 (13.8%)	80 (19.8%)
16 to 20 years	125(15.7%)	52 (13.2%)	73 (18.1%)
>20 years	301 (37.8%)	214 (54.5%)	87 (21.5%)
Undetermined	99 (12.4%)	52 (13.2%)	47 (11.6%)
**HLA-B27 positivity**			
	739 (92.7%)	380 (96.7%)	359 (88.9%)
**Baseline mSASSS at evaluati on**			
mSASSS <3	404 (50.7%)		
mSASSS <24	120 (15.0%)		
mSASSS ≤24	273 (34.3%)		

Sydesmophyte positive: AS patients with syndesmophyte formations (N = 393; mSASSS <24 plus mSASSS ≤24). Sydesmophyte negative, AS patients without syndesmophyte formations (N = 404; mSASSS <3)

mSASSS, modified Stoke's Ankylosing Spondylitis Spinal Score

### Association of ERAP1 SNPs with AS

Four ERAP1 SNPs (rs27037, rs27980, rs27044 and rs30187) were selected based on the SNP locations (chromosome position, coding and regulatory regions) and their functional relevance described in the previous studies. Those four SNPs were genotyped in 797 AS patients and 1,150 healthy controls. The distributions of ERAP1 SNP genotypes were conformed to the Hardy-Weinberg equilibrium in the genotyped subjects (*P *>0.05). As shown in Table 2, significant differences in the distributions of ERAP1 SNP genotypes and alleles were observed between AS patients and the normal healthy controls. Our analyses indicate that the SNP rs27037T allele is a risk factor for AS susceptibility (Trend test *P *= 4 × 10^-5 ^with 100,000 permutations; T vs. G, *P *= 5.5 × 10^-5^, OR 1.30, 95% CI: 1.15 to 1.48; genotypes GT+TT vs. GG, *P *= 9.3 × 10^-5^, OR 1.49, 95% CI: 1.22 to 1.82). On the other hand, the SNP rs27980C allele appears to be a modest risk factor for AS susceptibility (Trend test *P *= 0.035 with 100,000 permutations; C vs. A, *P *= 0.033, OR 1.15, 95% CI: 1.01 to 1.31; genotype AC+CC vs. AA, *P *= 0.024, OR 1.31; 95% CI: 1.04 to 1.65). In addition, both cSNP (coding SNP) rs27044G allele (Trend test *P *= 1.7 × 10^-4 ^with 100,000 permutations; G vs. C, *P *= 1.5 × 10^-4^, OR 1.28, 95% CI: 1.13 to 1.46; genotypes CG+GG vs. CC, *P *= 1.7 × 10^-3^, OR 1.44, 95% CI: 1.15 to 1.81), and the cSNP rs30187T allele (Trend test *P *= 1.6 × 10^-3 ^with 100,000 permutations. T vs. C, *P *= 1.7 × 10^-3^, OR 1.23, 95% CI: 1.08 to 1.40; genotype CT+TT vs. CC, *P *= 6.1 × 10^-3^, OR 1.38, 95% CI: 1.10 to 1.74) are risk factors for AS susceptibility.

**Table 2:** ERAP1 SNP analyses in Taiwanese AS patients and normal controls

SNP /Group	RA /RAF	Genotype /Frequency			Trend test P*	Genotype analysis		Allelic analysis	
						P	OR (95% CI)	P	OR (95% CI)
**rs27037**	**T**	**GG**	**GT**	**TT**			**GT + TT vs. GG**		**T vs. G**
Normal (N = 1150)	0.415	0.341	0.488	0.171	4 × 10^-5^	9.3 × 10^-5^	1.49 (1.22-1.82)	5.5 × 10^-5^	1.30 (1.15-1.48)
AS (N = 796)	0.481	0.258	0.524	0.219					
**rs27980**	**C**	**AA**	**AC**	**CC**			**AC + CC vs. AA**		**C vs. A**
Normal (N = 1150)	0.538	0.215	0.494	0.291	0.035	0.024	1.31 (1.04-1.65)	0.033	1.15 (1.01-1.31)
AS (N = 797)	0.573	0.173	0.508	0.319					
**rs27044**	**G**	**CC**	**CG**	**GG**			**CG + GG vs. CC**		**G vs. C**
Normal (N = 1149)	0.509	0.235	0.513	0.252	1.7 × 10^-4^	1.7 × 10^-3^	1.44 (1.15-1.81)	1.5 × 10^-4^	1.28 (1.13-1.46)
AS (N = 796)	0.570	0.176	0.508	0.317					
**rs30187**	**T**	**CC**	**CT**	**TT**			**CT + TT vs. CC**		**T vs. C**
Normal (N = 1150)	0.529	0.220	0.503 0.277		1.6 × 10^-3^	6.1 × 10^-3^	1.38 (1.10-1.74)	1.7 × 10^-3^	1.23 (1.08-1.40)
AS (N = 797)	0.580	0.169	0.502	0.329					

RA: Risk allele. RAF: Risk allele frequency. * The Trend test p-values were generated from 100,000 permutations.

### Association of ERAP1 SNPs with AS clinical parameters

AS is a heterogeneous chronic inflammatory disease and present with diverse clinical severity. Subsequently, we stratified AS patients according to clinical characteristics and analyzed the genetic data. As shown in Table 3, we observed that the cSNP rs27044G allele carriers (genotype CG+GG vs. CC, *P *= 0.015, OR 1.59, 95% CI: 1.33 to 2.30) and the cSNP rs30187T allele carriers (genotype CT+TT vs. CC, *P *= 0.011, OR 1.63, 95% CI: 1.12 to 2.38) are susceptible to syndesmophyte formation in AS patients. After adjusting for the effect of HLA-B27 positivity, the cSNP rs30187 remains significantly associated with syndesmophyte formation (CMH statistics = 4.236, degree of freedom = 1, *P *= 0.040) while cSNP rs27044 is marginally associated with syndesmophyte formation (CMH statistics = 3.843, degree of freedom = 1, *P *= 0.050). The remaining two SNPs did not show any significant associations. Because previous studies demonstrated that human leukocyte antigen HLA-B27 has a causative role in AS pathogenesis, we subsequently investigated whether there is interaction between the ERAP1 SNPs and HLA-B27 positivity. We observed that the distributions of genotypes of four ERAP1 SNPs revealed significant differences between AS patients positive for HLA-B27 and patients negative for HLA-B27 (Table 4). As shown in Table 4, allele distributions of three ERAP1 SNPs (rs27037, rs27044, and rs30187) were significantly different between AS patients positive for HLA-B27 and the patient negative for HLA-B27. Notably, two cSNPs (rs27044 and rs30187) are strongly associated with HLA-B27 positivity. The cSNP rs27044G allele was significantly enriched in AS patients positive for HLA-B27 (Trend test *P *= 6.3 × 10^-3 ^with 100,000 permutations; G vs. C, *P *= 5.8 × 10^-3^, OR 1.70, 95% CI: 1.16 to 2.49; genotypes CG+GG vs. CC, *P *= 7 × 10^-4^, OR 2.71, 95% CI: 1.53 to 4.82). The cSNP rs30187T allele was also significantly enriched in AS patients positive for HLA-B27 (Trend test *P *= 6.1 × 10^-3 ^with 100,000 permutations; T vs. C, *P *= 5.4 × 10^-3^, OR 1.71, 95% CI: 1.17 to 2.49; genotypes CT+TT vs. CC, *P *= 4 × 10^-4^, OR 2.86, 95% CI: 1.61 to 5.09). We also analyzed associations between ERAP1 SNPs and HLA-B27 positivity after controlling for the syndesmophyte formation. The CMH test results revealed that the significant associations between three ERAP1 SNPs (rs27037, rs27044, and rs30187) and HLA-B27 positivity (*P *<0.01). Our data indicate that interaction of ERAP1 and HLA-B27 may play a pivotal role in the pathogeneses of AS.

**Table 3:** ERAP1 SNP analyses in normal controls and AS patients positive and negative for syndesmophyte

SNP /Group	RA /RAF	Genotype /Frequency			Trend test P**	Genotype analysis		Allelic analysis	
						P	OR (95% CI)	P	OR (95% CI)
**rs27037**	**T**	**GG**	**GT**	**TT**			**GT + TT vs. GG**		**T vs. G**
Normal (N = 1150)	0.415	0.341	0.488	0.171	7 × 10^-5^	2 × 10^-4 ^	1.64 (1.27-2.14)	7.8 × 10^-5^	1.39 (1.18-1.63)
AS Syn+ (N = 393)	0.496	0.239	0.529	0.232					
AS Syn- (N = 403)	0.465	0.275	0.519	0.206	0.215	0.243	1.21 (0.88-1.66)	0.217	1.13 (0.93-1.38)
Adjusted for HLA-B27+*					0.338	0.449	1.13 (0.82-1.57)	0.351	1.10 (0.90-1.34)
**rs27980**	**C**	**AA**	**AC CC**				**AC + CC vs. AA**		**C vs. A**
Normal (N = 1150)	0.538	0.215	0.493	0.291	9.9 × 10^-3^	0.011	1.49 (1.10-2.02)	9.4 × 10^-3^	1.24 (1.05-1.46)
AS Syn+ (N = 393)	0.592	0.155	0.506	0.338					
AS Syn- (N = 404)	0.555	0.191	0.510	0.300	0.134	0.188	1.28 (0.89-1.85)	0.134	1.16 (0.95-1.42)
Adjusted for HLA-B27+					0191	0.311	1.21 (0.83-1.77)	0.195	1.14 (0.93-1.40)
**rs27044**	**G**	**CC**	**CG**	**GG**			**CG + GG vs. CC**		**G vs. C**
Normal (N = 1149)	0.509	0.235	0.513	0.252	7 × 10^-5^	10^-4^	1.85 (1.35-2.53)	9.8 × 10^-5^	1.38 (1.18-1.63)
AS Syn+ (N = 393)	0.589	0.143	0.537	0.321					
AS Syn-(N = 403)	0.552	0.208	0.479	0.313	0.136	0.015	1.59 (1.33-2.30)	0.136	1.16 (0.95-1.42)
Adjusted for HLA-B27+					0.271	0.050	1.46 (1.00-2.13)	0.277	1.12 (0.91-1.37)
**rs30187**	**T**	**CC**	**CT**	**TT**			**CT + TT vs. CC**		**T vs. C**
Normal (N = 1150)	0.529	0.220	0.503	0.277	1.3 × 10^-3^	3 × 10^-4^	1.81 (1.31-2.50)	1.5 × 10^-3^	1.31 (1.11-1.54)
AS Syn+ (N = 393)	0.594	0.135	0.542	0.323					
AS Syn-(N = 404)	0.566	0.203	0.463	0.334	0.258	0.011	1.63 (1.12-2.38)	0.248	1.12 (0.92-1.37)
Adjusted for HLA-B27+					0.463	0.040	1.50 (1.02-2.20)	0.456	1.08 (0.88-1.32)

RA: Risk allele. RAF: Risk allele frequency. The case-control association tests were performed in two subsets of the samples: (1) AS patients with syndesmophyte (AS Syn+) vs. normal controls; (2) AS patients with syndesmophyte (AS Syn+) vs. AS patients without syndesmophyte (AS Syn-). *Cochran -Mantel -Haenszel (CMH) tests for ERAP1 SNP and syndesmophyte associations, controlling for HLA-B27 positivity. ** The Trend test p-values were generated from 100,000 permutations.

**Table 4:** ERAP1 SNP analyses in normal controls and AS patients positive and negative for HLA-B27

SNP /Group	RA /RAF	Genotype /Frequency			Trend test P**	P	Genotype analysis OR (95% CI)	P	Allelic analysis OR (95% CI)
**rs27037**	**T**	**GG**	**GT**	**TT**			**GT + TT vs. GG**		**T vs. G**
HLA-B27 + (N = 738)	0.488	0.245	0.534	0.221	0.042	5.6 × 10^-3^	2.17 (1.26-3.76)	0.038	1.50 (1.02-2.21)
HLA-B27 - (N = 58)	0.388	0.414	0.397	0.190					
Adjusted for syndesmophyte+*					0.057	9.1 × 10^-3^	2.10 (1.20-3.66)	0.056	1.46(0.99-2.16)
**rs27980**	**C**	**AA**	**AC**	**CC**			**AC + CC vs. AA**		**C vs. A**
HLA-B27 + (N = 739)	0.578	0.165	0.514	0.321	0.172	0.034	1.93 (1.05-3.54)	0.147	1.32 (0.91-1.93)
HLA-B27 - (N = 58)	0.509	0.276	0.431	0.293					
Adjusted for syndesmophyte+					0.229	0.056	1.61 (0.99-3.38)	0.217	1.27 (0.87-1.86)
**rs27044**	**G**	**CC**	**CG**	**GG**			**CG + GG vs. CC**		**G vs. C**
HLA-B27 + (N = 738)	0.580	0.163	0.515	0.323	6.3 × 10^-3^	7 × 10^-4^	2.71 (1.53-4.82)	5.8 × 10^-3^	1.70 (1.16-2.49)
HLA-B27 - (N = 58)	0.448	0.345	0.414	0.241					
Adjusted for syndesmophyte+					0.010	2.7 × 10^-3 ^	2.45 (1.36-4.39)	0.011	1.64 (1.12-2.42)
**rs30187**	**T**	**CC**	**CT**	**TT**			**CT + TT vs. CC**		**T vs. C**
HLA-B27 + (N = 739)	0.589	0.156	0.510	0.334	6.1 × 10^-3^	4 × 10^-4^	2.86 (1.61-5.09)	5.4 × 10^-3^	1.71 (1.17-2.49)
HLA-B27 - (N = 58)	0.457	0.345	0.397	0.259					
Adjusted for syndesmophyte+					0.011	1.6 × 10^-3^	2.56 (1.43-4.61)	8.5 × 10^-3^	1.67 (1.14-2.45)

RA: Risk allele. RAF: Risk allele frequency. * Cochran -Mantel -Haenszel tests for ERAP1 SNP and HLA-B27 associations, adjusted by syndesmophyte formation. ** The Trend test p-values were generated from 100,000 permutations. Sydesmophyte +: syndesmophyte formation positive

### ERAP 1 SNP haplotypes are associated with AS susceptibility

**Figure 1:**
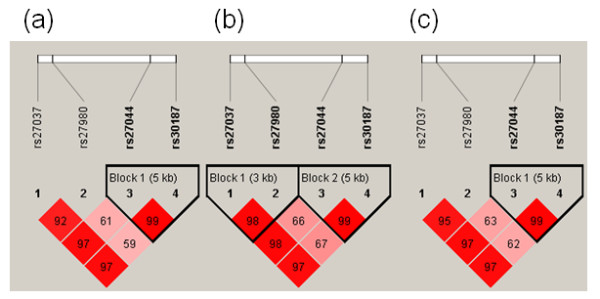
**Pairwise linkage disequilibrium (LD) plot patterns for four polymorphisms through ERAP1 regions on chromosome 5**. (**a**: controls N = 1,150; **b**: AS cases N = 797; **c**: Totals N = 1,947).

We next examined the influence of ERAP1 SNP haplotypes on AS susceptibility and syndesmophyte formation according to the linkage disequilibrium (LD) block (Figure 1). Due to the fact that two ERAP1 cSNPs (rs27044 and rs30187) are in extremely strong LD (0.99), we selected rs27044 as the representative marker for two cSNPs in the subsequent haplotypes analysis. As shown in Table 5, ERAP1 SNP haplotype TCG (rs27037T/rs27980C/rs27044G) frequency is significantly increased in AS patients (0.475) as compared to healthy controls (0.399) (adjusted *P *<0.00001, OR 1.38, 95% CI: 1.12 to 1.58), suggesting that the haplotype TCG is a risk marker for AS. On the other hand, haplotype GCC (rs27037G/rs27980C/rs27044C) frequency was significantly increased in healthy controls (0.119) compared to AS patients (0.085) (adjusted *P *= 1.9 × 10^-3^, OR 0.69, 95% CI: 0.55 to .87), indicating that the haplotype GCC has a protective role against AS. The haplotype TCG frequency is slightly higher in AS patients positive for syndesmophyte formation as compared to that in AS patients negative for syndesmophyte formation; however, no significant difference was observed (*P *>0.05)

**Table 5:** Haplotype analysis of ERAP 1 SNPs in 797 AS patients and 1150 normal controls.

Associated haplotypes	Frequency		EM algorithm*	Logistic regression		Logistic regression	
(rs27037 + rs27980 + rs27044)	AS	Normal	P -value	P -value	OR (95% CI)	(sex adjusted)	
						P -value	OR (95% CI)
GCC	0.085	0.119	9 × 10^-3^	7 × 10^-4^	0.69 (0.55-0.85)	1.9 × 10^-3^	0.69 (0.55-0.87)
TAG	0.008	0.012	2.6 × 10^-3^	3.9 × 10^-3^	0.21 (0.07-0.61)	4.3 × 10^-3^	0.21 (0.07-0.61)
TCG	0.475	0.399	<0.00001	<0.00001	1.36 (1.12-1.55)	<0.00001	1.38 (1.12-1.58)

*The p-values for the estimated haplotypes were generated from 10,000 permutations.

## Discussion

Complex interactions between environmental factors and host immune responses are the origins for AS development [4,5]. It is clear that genetic factors influence the immune responses and progression of AS. The current study demonstrated the associations of EARP1 SNPs with the AS susceptibility in Taiwanese. Aminopeptidases play a central role in the generation of MHC class I-binding peptides through processing and trimming peptide. As an IFNγ-induced aminopeptidase, ERAP1 breaks down protein antigen precursors and trims the peptides fragments into the suitable length for peptide/MHC I complex formation in ER [13,14,30]. In mice, ERAP1 deficiency led to the reduced MHC I expression in splenocytes and the decreased viral peptide presentation by MHC I on fibroblasts [31]. The generation of effective CD8 T cell responses was compromised in ERAP1 deficient mice as self and foreign antigen presentations were disrupted [31-36]. In humans, over-expression of ERAP1 was observed in AS patient dendritic cells (DCs) [37]. ERAP1 also enhances phagocytic activity of human macrophages through generating active peptides [38]. Functionally, ERAP1 may have a substantial role in the AS pathogenesis. Our current study demonstrated the associations of ERAP1 SNPs with the AS susceptibility in Taiwanese, indicating that ERAP1 has a role in AS development in Taiwanese

We observed that >90% of AS patients are HLA-B27 carriers. Our data confirmed that HLA-B27 plays the most critical role in AS disease progression. HLA-B27 may influence AS development through three possible mechanisms: I) HLA-B27 may preferentially bind distinctive arthritogenic peptides, II) inappropriately folded HLA-B27 heavy chain without a docking antigen peptide may lead to the unfolded protein responses (UPRs), and III) HLA-B27 may have a tendency to be expressed as empty MHC I heavy chain homodimers [4,39]. However, the precise mechanisms underlying the critical role of HLA-B27 in AS development remain to be elucidated [40].

Of great importance, immune surveillance of the CD8 T cell to environmental insults, such as bacterial and viral infections, requires the interaction between T cell receptor (TCR) and peptide/MHC I complex. ERAP1 trims protein antigens to fit for MHC I including HLA-B27. The stability of antigen peptide/MHC I complexes is influenced by both ERAP1 activities and peptide binding groove sequences of MHC I [41]. Very recently, Evans *et al*. demonstrated the gene-gene interaction between ERAP1 SNP rs30187T allele and HLA-B27 positivity in the pathogenesis of AS [42]. Our study provided further evidence that ERAP1 SNPs are indeed associated with the HLA-B27 positivity in Taiwanese AS patients. Our data support the notion that ERAP1 and HLA-B27 have synergistic roles in AS pathogenesis in humans. Our findings also suggest that abnormal antigen processing by ERAP1 and antigen presentation by HLA-B27 may be critical pathways in AS development. On the other hand, AS patients negative for HLA-B27 may develop pathologic immune responses through the other unidentified biological pathways.

The disease course of AS is heterogeneous and the genetic factors are believed to influence disease susceptibility and severity. Early syndesmophyte formations in AS patients indicate radiologic progression and characteristic structure changes that lead to the decreased spinal mobility, functional impairments and work disability in long-term disease courses [4,43]. Sacroiliac joint biopsy examinations frequently revealed significant new bone formations and bony ankylosis in AS patients [44]. Spinal inflammation and bone remodeling may be two important factors in the progress of ankylosis but the precise pathogenesis of AS remains unknown [44,45]. The current study demonstrated that ERAP1 cSNPs rs27044 and rs30187 are modestly associated with AS syndesmophyte formation, suggesting that the ERAP1 cSNPs may affect AS disease severity. Syndesmophyte of AS is more likely to develop at sites of previous inflammation, indicating ankylosis development is strongly correlated with inflammation [44]. The ERAP1 protein also participates in the regulation of proinflammatory cytokine receptor functions. ERAP1 cleaves membrane-associated TNFR1, IL-6R, and interleukin 1 receptor II (IL-1RII) and causes the shedding of those cytokine receptors. Some cellular and molecular signal pathways that regulate the development of hematopoietic cell and bone homeostasis are shared by the immune system and bone development [45]. IL6 and TNFα cytokine networks may affect the Th17 cell development and the plasticity of T cell differentiation, which are critical in the pathogenesis of AS. The serum cytokine receptor levels in AS patients are correlated with the levels ESR and CRP, which are indicators of AS inflammatory activities. Nevertheless, the serum cytokine receptor levels in patients with AS are not influenced by ERAP1 SNPs [46]. Animal models have even suggested that inflammation and new bone formation are uncoupled processes [47,48]. Clinically, various anti-TNF therapies suppress the inflammation process but do not retard the structural progression according to modified Stoke's Ankylosing Spondylitis Spinal Score (mSASSS) [49-51]. These findings indicate the syndesmophyte development may largely attribute to the intrinsic genetic effects of ERAP 1 on p/MHC I complex formation.

UPRs can cause the activation of NFκB, which could enhance downstream proinflammatory gene expression and promote inflammations. Genetic variations of HLA-B27 and ERAP1 have functional roles in the misfolding and UPR of the heavy chain [4,40]. HLA-B27 misfolding is triggered in the oxidizing environment in endoplasmic reticulum (ER) by exposing cysteine residues within the heavy chain [40]. ERAP1 is required for efficient enzymatic activities that suggest the polymorphisms at sites remote from the catalytic sites might modify this association [52]. ERAP1 processes peptide substrates with the optimal sizes for MHC I (40, 41). Crystallography analyses suggest that the coding SNP rs30187 changes the amino acid residue that may affect the catalytic activity [53]. Notably, the alleles and genotypes of ERAP1 SNPs in Taiwanese and other Asians showed different distribution and opposite risk results in AS susceptibility compared to Caucasians [9-12,21-26]. In addition, ERAP1 SNP rs30187C allele carriers (CC+CT) were found to be associated with higher baseline radiographic severity based on mSASSS on univariate analysis [54]. The current study demonstrated that both SNP rs30187T and rs27044G allele carrier were modestly associated with AS disease severity of syndesmophyte formation. Functional studies of ERAP1 SNPs have identified that the rs30187C allele carries less biological enzyme activity, which may decrease aberrant peptide processing and HLA-B27 presentation [42], the rs27044C allele carrier genotype demonstrated significantly higher free heavy chain (FHC) expression but lower intact HLAB27 complexes/FHCs ratio [55], and ERAP1 SNPs (rs30187 and rs27044) showed specific peptide substrate sequence interaction [52]. Moreover, large multifunctional peptidase 2 (LMP2) rs17587 SNP has been demonstrated to associate with AS radiographic severity [54]. These findings indicated multiple gene interactions are involved in the complexity of AS disease susceptibility and severity, and no definite functional roles of ERAP1 cSNPs rs30187 and rs27044 in antigen processing can fully answer the discrepancy results in different ethnic backgrounds. Nevertheless, other ERAP1 SNPs may also affect UPR and subsequent immune responses. Further functional studies are required to understand the precise roles of ERAP1 SNPs contribute to the AS pathogenesis.

In this cross sectional study, some AS patients may not receive enough follow-up in the syndesmophyte formation assessment, which is the main limitation of our study. Therefore, future longitudinal studies are required to estimate the effect of ERAP1 SNPs on the bamboo spine development in large AS patient cohorts.

## Conclusion

This study provided further evidence that ERAP1 interaction with HLA-B27 is involved in the development of AS and disease severity, which emphasized the critical role of pMHC I pathway genes in the pathogenesis of AS. Identification of the functional causal alleles of ERAP1 provides a new avenue in understanding the molecular mechanisms of AS pathogenesis, which may lead to novel treatment approaches.

## Abbreviations

AS: ankylosing spondylitis; CMH: Cochran -Mantel -Haenszel; DCs: dendritic cells; DM: diabetes mellitus; ER: endoplasmic reticulum; ERAP1: endoplasmic reticulum aminopeptidase 1; FHC: free heavy chain; GWAS: genome wide association studies; IL: interleukin; LD: linkage disequilibrium; LMP2: large multifunctional peptidase 2; MHC: major histocompatibility complex; mSASSS: modified Stoke's Ankylosing Spondylitis Spinal Score; ORs: odds ratios; RA: rheumatoid arthritis; SLE: systemic lupus erythematous; SNP: single nucleotide polymorphism; TCR: T cell receptor; TNFR1: tumor necrosis factor receptor 1; UPRs: unfolded protein responses

## Competing interests

The authors declare that they have no competing interests.

## Authors' contributions

CMW and JYC carried out the design of the study and participated in statistical analysis and manuscript writing. HHH performed clinical evaluation of the patients and participated in the design of the study. SWC performed statistical analysis. YJJW and JCL participated in clinical evaluation of the patients. PYC helped on laboratorial determination. JW participated in the design of study and manuscript revision. All authors read and approved the final manuscript.

## Acknowledgements

The authors would like to thank Shin Chu Blood Donor Center for sample collection. This study was supported by grants from Chang Gung Memorial Hospital (No. CMRPG381082) and National Science Council, Taiwan (NMRPG-97-2314-B182A-025-MY3).
